# Fermented palm kernel cake improves the nutrient degradation of beef cattle by modulating the rumen microbiota

**DOI:** 10.3389/fmicb.2025.1712275

**Published:** 2025-12-11

**Authors:** Hui Mi, Zhaozhe Wang, Haixiang Xiao, Jinjia Zhu, Pengxia Hou, Chuanshe Zhou, Dingfu Xiao

**Affiliations:** 1 College of Animal Science and Technology, Hunan Agricultural University, Changsha, China; 2 Yuelushan Laboratory, Changsha, China; 3 State Key Laboratory of Forage Breeding-by-Design and Utilization, National Engineering Laboratory for Pollution Control and Waste Utilization in Livestock and Poultry Production, and Hunan Provincial Key Laboratory of Animal Nutritional Physiology and Metabolic Process, Institute of Subtropical Agriculture, Chinese Academy of Sciences, Changsha, Hunan, China; 4 Institute of Animal Science, Ningxia Academy of Agricultural and Forestry Sciences, Yinchuan, China

**Keywords:** palm kernel cake, solid state fermentation, beef cattle, growth performance, rumen

## Abstract

**Introduction:**

This study aimed to evaluate the effects of fermenting palm kernel cake (PKC) using Aspergillus niger and mannanase on its nutritional quality, and to assess the impact of replacing soybean meal and corn with fermented PKC (FPKC) on growth performance, rumen fermentation, nutrient digestibility, and rumen microbiota in beef cattle.

**Methods:**

PKC was co-fermented with Aspergillus niger and mannanase to enhance its nutritional profile. Thirty beef cattle were randomly assigned to two dietary groups (n = 15 per group): a control group (CON) receiving a conventional diet, and a treatment group (FP) fed a diet in which soybean meal and corn were partially substituted with FPKC. A 90-day feeding trial was conducted, during which body weight was monitored, and rumen fluid and fecal samples were collected on days 30, 60, and 90 to evaluate growth performance, rumen fermentation, and nutrient digestibility. On day 90, rumen samples were subjected to 16S rRNA gene sequencing to characterize the rumen microbial community composition.

**Results:**

Co-fermentation significantly improved the nutritional quality of PKC and reduced its mannan content (*P* < 0.05). Although growth performance did not differ significantly between the FP and CON groups throughout the trial (*P* > 0.05), the FP group exhibited significantly higher digestibility of dry matter (DMD), crude protein (CPD), and neutral detergent fiber (NDFD) after 90 days compared to the CON group (*P* < 0.05). Rumen fermentation analysis revealed lower concentrations of butyrate and NH_3_-N in the FP group than in the CON group (*P* < 0.05), while other fermentation parameters showed no significant differences (*P* > 0.05). 16S rRNA sequencing indicated that the FP group had a higher relative abundance of Bacteroidetes, whereas Firmicutes were more prevalent in the CON group. The *Rikenellaceae RC9 gut* group was identified as a dominant genus in the FP group, while the CON group was characterized by higher abundances of *Ruminococcus*, *Christensenellaceae R-7* group, and *Butyrivibrio*.

**Conclusion:**

These findings demonstrate that co-fermentation with Aspergillus niger and mannanase effectively enhances the nutritional value of PKC. The inclusion of FPKC in the diet improves nutrient digestibility and modulates rumen microbial composition in beef cattle, supporting its potential as a valuable alternative feed ingredient.

## 1 Introduction

The world population has been continuously growing and is anticipated to reach up to 9.8 billion by 2050 (United Nations, 2017). Given the enormous growth of the population, the need for animal products will need to expand (Devendra and Leng, 2011). However, animal production is under restriction because of inadequate feed supply caused by rapid urbanization and industrialization (Saritha et al., 2012; Röös et al., 2017). Moreover, the competition for available resources (food or land) between humans and animals is a growing challenge. Therefore, to achieve the desired animal production level, finding efficient and sustainable non-grain feed ingredients for animal nutrition is necessary (Mahesh, 2014).

Globally, plant by-products (PBP) are considered to be important feed resources that are not edible for humans and can be used in livestock production (Salami et al., 2019). Palm kernel cake (PKC) is an agro-industrial by-product of oil palm production, and due to its essential amino acid content and high protein composition, it can be a viable raw material for replacing protein ingredients or as a feed supplement (Kari et al., 2022). However, the PKC contains a large amount of crude fiber and high concentrations of NSP, primarily *β*-mannan and cellulose (Olukomaiya et al., 2019). Due to beta-mannan mixed with saliva, it will become a sticky solution, which will cause uneven mixing of various components in chyme, resulting in reduced contact between endogenous digestive enzymes and nutrients, and ultimately affect the digestibility and production performance of animal nutrients (Chang-qing et al., 2019).

In recent years, researchers have increasingly focused on using fermentation technology and enzymatic hydrolysis to enhance feed nutrition and livestock product yield (Sugiharto and Ranjitkar, 2019; Aguzey et al., 2020; Su et al., 2020; Yang et al., 2021). The synergistic application of enzymatic hydrolysis and microbial fermentation improves the conversion of complex substances and anti-nutrients in feed into accessible and safe nutrients (Cao et al., 2024; Liu et al., 2025). Therefore, to address the issue of high NSP content in PKC, this study implemented a co-fermentation strategy with mannanase and *Aspergillus niger* (a cellulase producer) on PKC, and incorporated the fermented PKC in beef cattle diets (Yao et al., 2015). The investigation assessed the impact of FPKC on the growth performance, nutrient digestibility, and rumen fermentation patterns in beef cattle. The findings elucidated the potential of FPKC as a viable feed ingredient in animal production and offered novel insights into the efficient utilization of agricultural by-products.

## 2 Method

### 2.1 Synergistic fermentation of PKC by bacteria and enzymes

#### 2.1.1 Ingredients and strains

PKC was purchased from Hunan Minchu Biotechnology Co., LTD. The *Aspergillus Niger* was provided by Shenzhen Saiyiyang Biotechnology Co., LTD. Mannanase (50,000 U/g) was provided by Beijing Challenge Biotechnology Co., LTD.

#### 2.1.2 Suitable concentration of PKC fermented by bacteria and enzymes

Using *in vitro* solid fermentation technology, a single-factor experimental design was used to select mannanase and *Aspergillus Niger* to ferment PKC. 6 additional levels of 0, 0.1, 0.2, 0.3, 0.4 and 0.5% were designed, respectively, and three replicates were set for each level. After the mixture was uniform, 100 g of the sample was transferred to a vacuum bag, sterile water was added to adjust the moisture content to approximately 50%, and the bag was sealed with a vacuum sealer. Anaerobic fermentation was carried out at room temperature for 36 days. After the fermentation is completed, the FPKC is collected for subsequent analysis.

#### 2.1.3 Effect of bacterial and enzyme co-fermentation of PKC

The optimal addition level of mannanase and *Aspergillus Niger* was selected, and the combination of mannanase and *Aspergillus Niger* was used for *in vitro* solid fermentation of PKC. The fermentation humidity and duration are consistent with those in 2.1.2. After the fermentation is completed, the FPKC is collected for subsequent analysis.

#### 2.1.4 FPKC sample collection and analysis

Upon completion of the fermentation process, the PKC was fermented with mannanase, *Aspergillus Niger*, and a mixture of mannan and *Aspergillus Niger* is dried and subjected to drying in an oven at 65 °C for 72 h, followed by grinding to a 40-mesh size. Nutrient composition, including dry matter (DM) and crude protein (CP), was analyzed per Association of Official Analytical Chemists International (AOAC) standards (AOAC, 2006). Neutral detergent fiber (NDF) and acid detergent fiber (ADF) were measured using a fiber analyzer (A200i, ANKOM, NY, United States) as described by Van Soest et al. (1991), and mannan content was assessed with a mannan ELISA detection kit (Thermo Fisher Scientific, Wilmington, DE, United States).

### 2.2 Effect of FPKC on the growth and rumen fermentation of beef cattle

#### 2.2.1 Animal management and dietary treatments

In this study, thirty healthy fattening cattle with an average initial body weight of 292.39 ± 35.44 kg were utilized. The cattle were stratified based on initial body weight (IBW) and subsequently randomized into two groups, each comprising 15 cattle. Groups were randomly assigned to one of the following two diets: (1) the control group (CON) was fed the basic diet following the “Beef Cattle Feeding Standards” (NY/T815-2012), (2) the experimental group (FP) was provided with a diet where fermented palm kernel meal replaced a portion of corn and soybean meal and under the condition of equal nitrogen. The formulation of the experimental diets was based on the NRC (2012) standards, with the basal diet composition and nutritional levels presented in Supplementary Table S1. The livestock facility was adequately ventilated, and before the commencement of the experiment, the flooring, walls, and enclosures of the cattle housing were meticulously disinfected. During the pre-feeding phase, the health status of the cattle was assessed, and all cattles underwent antiparasitic treatment.

Each group of cattle underwent a 14-day adaptation period, followed by a formal experimental phase lasting 3 months. During the experimental period, the animals were provided with their respective diets twice daily at 06:30 and 16:00, with unrestricted access to water.

#### 2.2.2 Growth performance

Throughout the duration of the experiment, the daily feed intake was meticulously recorded to ascertain the average daily feed intake (ADFI). Concurrently, the experimental beef cattle were weighed at intervals on days 0, 30, 60, and 90 to determine the average daily gain (ADG). Furthermore, the feed conversion ratio (F/G) was calculated utilizing the ADFI and ADG.

#### 2.2.3 Fecal sample collection and apparent nutrient digestibility analysis

Samples of diet and feces of each group were obtained at 30 d, 60 d, 90 d, then stored at −80 °C for subsequent analysis. The determination of nutritional composition in feed and fecal samples is as described in the determination method of fermented PKC. Furthermore, acid-insoluble ash (AIA) served as an endogenous marker for determining the apparent digestibility of nutrients. The digestibility was calculated by the following formula:


digestibility(%)=(100−A1/A2×F2/F1×100)

A1 and A2 are the AIA contents of the feed and feces, respectively, and F1 and F2 are the nutrient contents of the feed and feces, respectively (Zhu et al., 2022).

#### 2.2.4 Rumen fluid collection and analysis

The time points for collecting rumen fluid were consistent with those for collecting feces samples (30 d, 60 d, 90 d). Ruminal samples were obtained utilizing an oral stomach tube. To reduce saliva contamination, the initial 50 mL of ruminal fluid was discarded. Subsequently, the rumen fluid samples were collected by filtering the ruminal contents through sterile four-layer cheesecloth. Upon extraction from the rumen, a 5 mL aliquot of the rumen sample was promptly frozen in liquid nitrogen and subsequently stored at −80 °C to inhibit microbial activity for subsequent DNA extraction. The ruminal pH was measured immediately following sample collection using a portable pH meter (Starter 300; Ohaus Instruments Co. Ltd., Shanghai, China). The concentration of volatile fatty acids (VFA) was quantified via gas chromatography, following the methodology outlined by Harvatine et al. (2002). The concentration of ammonia nitrogen (NH_3_-N) was assessed using the phenol-hypochlorite reaction method as described by Broderick and Kang (1980). Microbial protein (MCP) concentration was measured employing the Coomassie Brilliant Blue G-250 staining technique.

#### 2.2.5 DNA extraction and 16S rRNA gene sequencing

The microbial DNA from the rumen was extracted utilizing the E.Z.N.A. DNA kit (Omega Biotek, Norcross, GA, United States) in accordance with the manufacturer’s instructions. DNA concentration was quantified using a Nanodrop-2000 spectrophotometer (Thermo Fisher Scientific, Wilmington, DE, United States), and the integrity of the DNA was evaluated via 1% agarose gel electrophoresis. Amplification of the bacterial 16S rRNA gene fragments (V1–V9) was performed using the forward primer 27F (5’-AGRGTTYGATYMTGGCTCAG-3′) and the reverse primer 1492R (5’-RGYCCTTTGTTACGACTT-3′). The PCR products were visualized on 2% agarose gels and subsequently purified using the QIAquick gel extraction kit (Qiagen, Dusseldorf, Germany). Sequencing was conducted on an Illumina MiSeq PE300 paired-end platform by Shanghai Lingen Biomedical Technology Co., Ltd. (Shanghai, China). The sequences were analyzed utilizing QIIME 2^1^ with default settings. Operational taxonomic units (OTUs) were clustered using UPARSE, applying a 97% similarity threshold. All representative sequences were annotated against the Silva database (version 123) employing the RDP classifier. Taxonomic identification and comparative analyses were conducted at both the phylum and genus levels. Alpha diversity indices, including Chao1 and Shannon, were computed using QIIME2 with its default parameters. The raw data were uploaded to the NCBI SRA database (BioProject ID: PRJNA1294514). The microbial community structures were analyzed through Principal Coordinate Analysis (PCoA) employing the weighted UniFrac distance metric. To identify bacterial taxa exhibiting significant differences in relative abundance across domain to genus levels between the two groups, the Linear Discriminant Analysis Effect Size (LEfSe) method (accessible at http://huttenhower.sph.harvard.edu/LEfSe) was utilized, with criteria set at LDA > 4 and *p* < 0.05. To further quantify group differences in relative abundance, we compared the relative abundances of LEfSe-identified taxa between the two treatment groups using the Wilcoxon rank-sum test.

### 2.3 Statistical analysis

Statistical analyses to assess differences between the two groups were conducted using independent-samples *t*-tests in SPSS version 22.0. Data are presented as mean ± standard error of the mean, with statistical significance established at *p* < 0.05.

## 3 Result

### 3.1 Effects of co-fermentation of bacteria and enzymes on the nutritional characteristics of PKC

**Figure 1:**
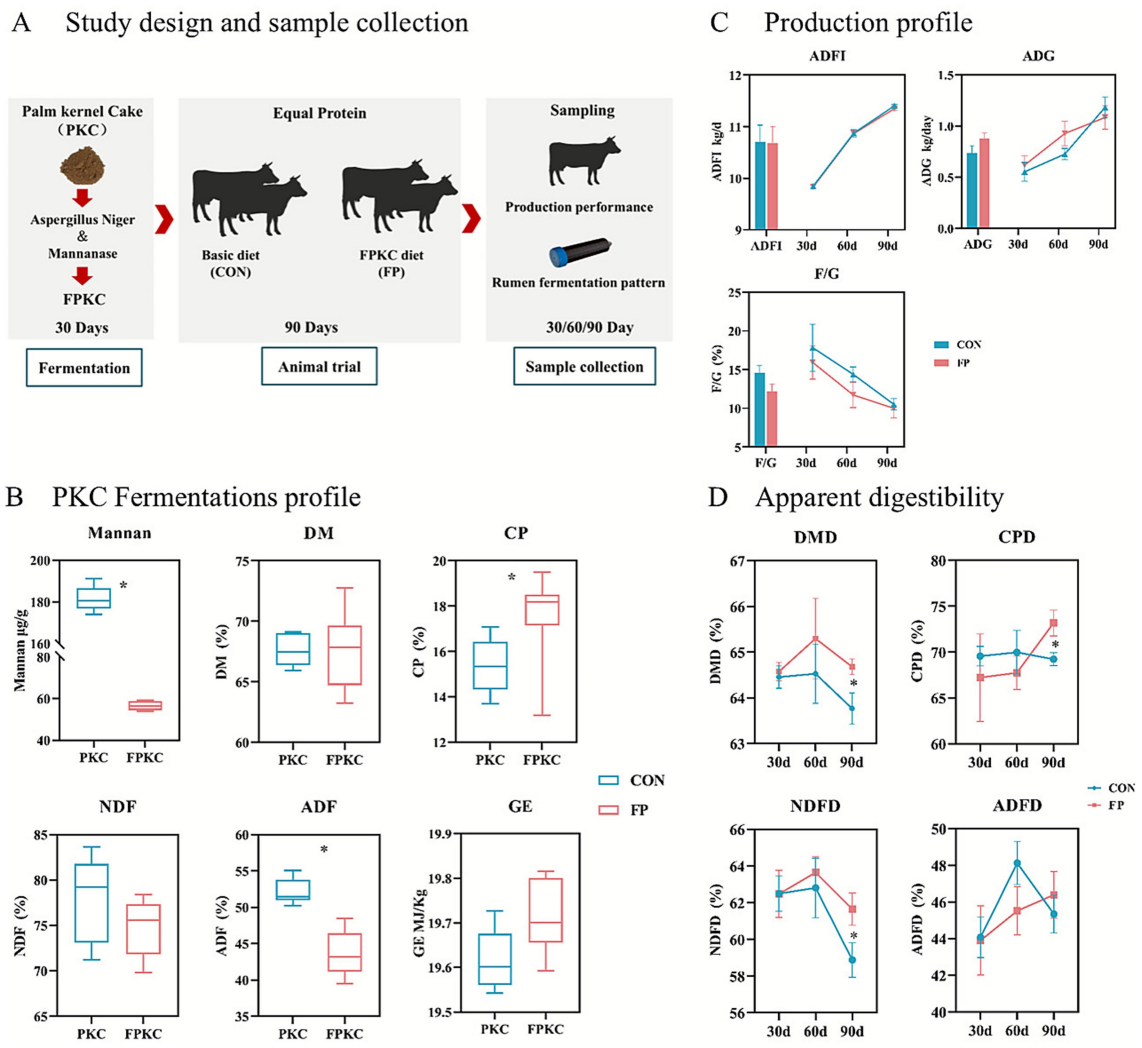
The nutritional composition of FPKC and its impact on the production efficiency and digestive metabolism in beef cattle. **(A)** Overview of design and sample collection. **(B)** PKC Fermentations profile. **(C)** Production profile. **(D)** Nutrient apparent digestibility. CON, the control group; FP, FPKC diet. Asterisks denote significant *p*-values, *p < 0.05.

The selection of the appropriate concentration of PKC fermented by *Aspergillus Niger* and mannanase is mainly based on the mannan concentration (Figure 1A). The research results show that the mannan concentrations of PKC fermented with 0.5% concentration of mannan (Supplementary Table S2) and 0.1% concentration of *Aspergillus Niger* (Supplementary Table S3) are the lowest, which are 82.42 μg/g and 51.60 μg/g, respectively. Based on this, as shown in Figure 1B, co-fermentation of mannan and *Aspergillus niger* can significantly reduce the mannan concentration and ADF content of PKC, and it has significantly improved CP content (*p* < 0.05).

### 3.2 Effect of FPKC on the production performance and nutrient apparent digestibility of beef cattle

In terms of production performance, no significant differences were observed in ADFI, ADG or F/G between the FP and CON during the test period (Figure 1C). In terms of the apparent digestibility, FP group exhibited significantly higher levels of dry matter digestibility (DMD), crude protein digestibility (CPD), and neutral detergent fiber digestibility (NDFD) compared to the CON group after 90 days of the experiment (*p* < 0.05) (Figure 1D).

### 3.3 Effect of FPKC on the beef cattle rumen fermentation parameters

**Figure 2:**
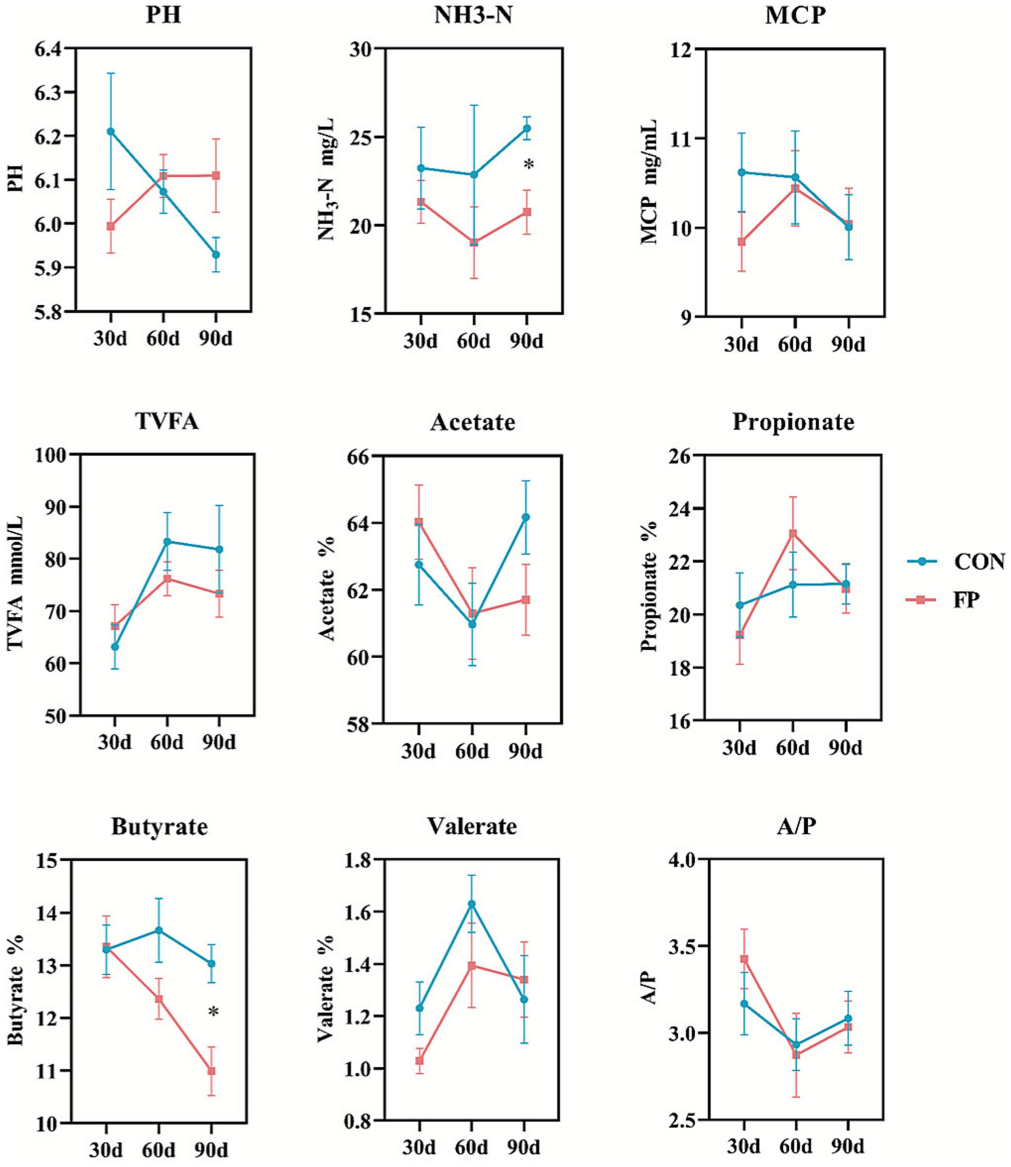
Fermentation indicators profiles in the rumen. CON, the control group; FP, FPKC diet. Asterisks denote significant *p*-values, *p < 0.05.

As shown in Figure 2, the two groups of cows did not differ in any of the determined rumen fermentation characteristics at the first 2 months (0-60d), but FP significantly decreased in NH_3_-N and Butyrate after 90 days of the experiment compared with the CON (*p* < 0.05).

### 3.4 Effect of FPKC on the rumen bacterial community of beef cattle

**Figure 3:**
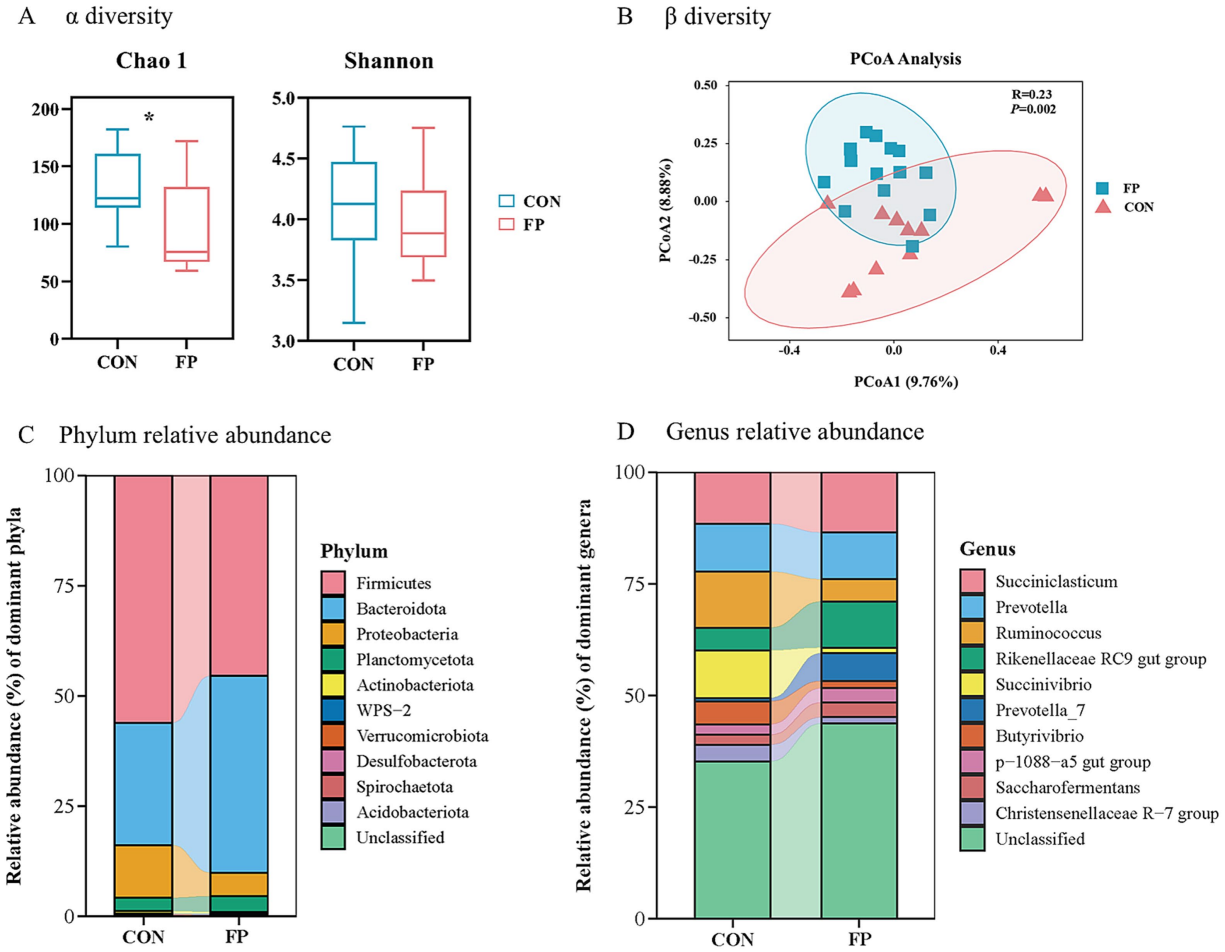
The impact of the FPKC diet on the microbial composition within the rumen of beef cattle. **(A)** *α* diversity. **(B)** *β* diversity, principal coordinate analysis (PCoA). **(C)** Relative abundances of the top 10 phylum. **(D)** Relative abundances of the top 10 genus. CON, the control group; FP, FPKC diet. Asterisks denote significant *p*-values, *p < 0.05.

**Figure 4:**
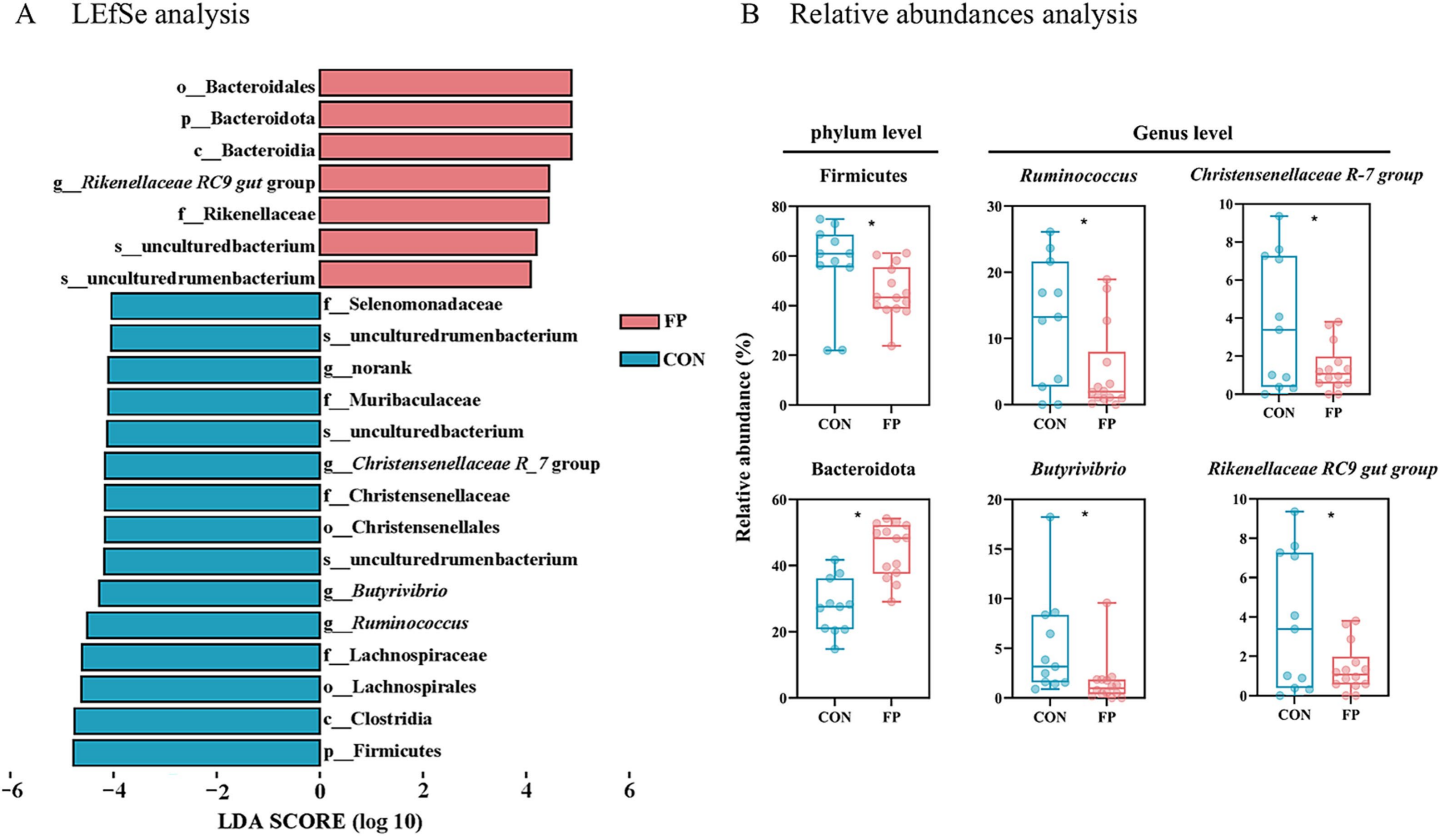
LEfSe analysis of bacterial taxa distinguishing CON and FP groups. **(A)** Linear discriminant analysis effect size (LEfSe) between the CON and FP groups. **(B)** Boxplots of the relative abundance of key bacterial identified by LEfSe analysis. Statistical significance is indicated with *p*-values using Wilcoxon test for each comparison. CON, the control group; FP, FPKC diet. Asterisks denote significant *p*-values, *p < 0.05.

The CON group have significantly greater Chao 1 values than the FP group (*p*  <  0.05) (Figure 3A). The PCoA and ANOSIM (R = 0.14, *p* = 0.002) showed a significant difference in the beta diversity between these two groups (Figure 3B). The distribution of species at the phylum level revealed that most of the annotated phylum was Firmicutes followed by Bacteroidetes (Figure 3C). At the genus level, *Prevotella* and *Succiniclasticum*, were the first two abundant genus (Figure 3D). There have 7 taxa identified by LEfSe enriched in the FP group (Figure 4A) (*p* < 0.05, LDA > 4). Conversely, 15 taxa enriched in CON (Figure 4A) (*p* < 0.05, LDA > 4). Among that, the relative abundance of *Ruminococcus*, *Butyrivibrio*, and *Christensenellaceae R-7* group was greater in the CON group at the genus level (*p* < 0.05) (Figure 4B). On the other hand, the relative abundance of *Rikenellaceae RC9 gut* group was notably higher in the FP group at the genus level (*p* < 0.05) (Figure 4B).

## 4 Discussion

PKC represents a feasible alternative for incorporation into cattle feed, offering potential reductions in production costs. However, the high content of mannan, an anti-nutritional factor, is the main factor restricting its wide application (Azizi et al., 2021). The degradation of mannan is predominantly facilitated by fungal species, particularly those belonging to the genus Aspergillus (Ong et al., 2024). Therefore, we chose to use *Aspergillus niger* and mannanase for the PKC fermentation study. The results demonstrated that the incorporation of either *Aspergillus niger* or mannanase effectively reduces mannan content, with 0.1% *Aspergillus niger* and 0.5% mannanase exhibiting the most pronounced effects. Based on this result, we combined *Aspergillus niger* and mannanase at the optimal concentration for PKC fermentation. The results showed that co-fermentation not only reduced the contents of mannan and ADF, but also increased the CP content of PKC. This agrees with the conclusions from previous studies that FPKC contains low hemicellulose and cellulose concentration but high protein content (Srivastava et al., 2018; Lee et al., 2019). Briefly, the co-fermentation process involving *Aspergillus niger* and mannanase significantly enhances the overall nutritional quality of PKC.

To further explore the feasibility of the FPKC as a feed ingredient for beef cattle, a study on the formulation of beef cattle diets using FPKC and subsequent feeding trials was conducted. Between groups, our study reveals that for production performance, there are no significant differences. Similarly, it was reported that adding 3% FPKC to cattle’s standard farm diet did not affect the ADG or F/G of beef cattle (Jiang et al., 2024). Moreover, it has been reported that ADG and F/G were not altered when cattle were fed diets with 8–24% PKC (de Melo Lisboa et al., 2020). Therefore, it suggests that a certain proportion of FPKC incorporation in the diets of cattle would not affect the production performance.

The apparent digestibility of nutritional components is an important characteristic of the diet quality. In the present study, the apparent digestibility for DM, CP, and NFD of the FP group was significantly higher than that in the CON group after 90 days of feeding. It is worth noting that in previous studies on PKC, due to its high mannan and fiber content (as ADF), adding PKC to formulate diets did not have a positive impact on the digestion of nutrients in animals (da Conceição dos Santos et al., 2016; Ferreira et al., 2022; Filho et al., 2024). Therefore, the higher digestibility of nutrients in the FP diet compared to the CON group might be due to the fact that the mannan and fiber content were reduced after co-fermentation by PKC, making it more easily digestible. In addition, a large number of studies have shown that bacteria and enzymes in fermentation feed can change the colonization and fermentation patterns of bacteria in the digestive tract, thereby improving the digestive process (Gomaa and Gado, 2021; Mousa et al., 2022; Sayed et al., 2024).

We speculate that FPKC may alter the rumen fermentation pattern, thereby contributing to improved digestion. It is well recognized that the significance of rumen microbiota lies in its critical role in converting feed into nutrients, with NH_3_-N, MCP, and VFA as key bacterial metabolites (Koh et al., 2016; Steele et al., 2016). NH_3_-N concentration was decreased, and the MCP yield was not affected by the FP group in the present experiments, which manifested late in the feeding period. The concentration of NH₃-N in the rumen reflects the proteolytic release and microbial N utilization and synthesis into MCP (Genzebu and Weldeslasse, 2015). Previous studies have shown that an optimal concentration of NH_3_-N ranges from 15 to 30 mg/dL, which can enhance animal feed intake, MCP synthesis, and nutrient digestibility, whereas NH_3_-N deficiency inhibits bacteria growth rate (Wanapat and Pimpa, 1999; Firkins et al., 2007). Both our experiment groups, NH_3_-N, were well within the normal range; thus, MCP synthesis is not affected in these cattle. On the basis of the comparative analysis of TVFA, it can be found that, for the two groups, the significant difference appears in the Butyrate components on 90 days, but the total VFA (TVFA) and other VFAs concentrations do not show a difference. VFAs produced in the rumen are affected by several factors, such as pH, feed composition, and microbial species (Atasoy et al., 2019). The observed reduction in butyrate within the FP group corroborates the findings of Zhang et al. (2025), which suggest that a diet characterized by slow-fermentation fiber results in diminished rumen butyrate levels. Accordingly, the results of the current study may be attributed to the replacement of corn and soybean meal with FPKM, thereby transitioning the diet from “high-starch, fast-fermentation” to “high-fiber, slow-fermentation,” and altering the rumen microbial hydrogen metabolism (Li et al., 2022).

Based on the above results, we further investigated the impact of our intervention on rumen microbiota composition. The index Chao1 reflects species richness, whereas the Shannon index indicates both richness and evenness. The results showed significant differences in Chao1 between the FP and CON groups, with the CON group exhibiting higher bacterial richness. Meanwhile, another trial of *β* diversity analysis revealed that the distribution of samples between the two groups was significantly different. These results showed that the rumen microbiota of the FP diet changed significantly. In order to further clarify the microbial communities of groups CON and FP, the OTUs were classified at the phylum and genus levels. The results of phylum level analysis showed that Firmicutes and Bacteroidetes were the dominant phyla in each group. This observation aligns with the findings that the predominant microbial communities within the cattle rumen remain unaffected by variations in dietary structure (Zhu et al., 2021; Sim et al., 2022). The FP group had a higher Bacteroidetes content, which aids in breaking down proteins, carbohydrates, and fibers in feed (Jiang et al., 2024). This suggests that substituting part of the diet with fermented palm kernel cake improved the cattle’s ability to decompose fibrous substances and CP, explaining the higher CPD and NDFD in the FP group. At the genus level, the relative abundance of *Rikenellaceae RC9 gut* group in the rumen was significantly higher in the FP group than in the CON group. The *Rikenellaceae RC9 gut* group, a member of the Rikenellaceae family, demonstrates the capability to efficiently degrade both soluble polysaccharides and insoluble cellulose, thereby contributing substantially to rumen health (Koike et al., 2015). It has been established that an increase in its relative abundance can improve the NDFD in feed, which is consistent with our findings (Wang et al., 2019; Zhang et al., 2021). On the other hand, the FP group had significantly lower *Ruminococcus*, *Christensenellaceae R-7* group and *Butyrivibrio* abundance than the control group. *Ruminococcus* has strong amylase activity (Liu et al., 2024). The elevated fiber content in the FP diet might be the reason for the observed decrease in rumen *Ruminococcus*. *Christensenellaceae_R-7* group, a member of the Firmicutes phylum, plays a significant role in the degradation of cellulose and hemicellulose within the rumen (Dai et al., 2015). The relative abundances of *Christensenellaceae_R-7* group were significantly higher in the CON group than in the FP group, consistent with the findings of Jiang et al. (2024). *Butyrivibrio* represents a crucial genus for the production of butyrate (Dai et al., 2021). In this experiment, the relative abundance changes of *Butyrivibrio* between the two groups were consistent with the rumen fermentation results. In conclusion, the findings of this study suggest that FPKC may enhance feed utilization by diminishing anti-nutritional factors and modifying microbial colonization and fermentation patterns. While FPKC shows potential as an alternative feed source, it is important to note that this study primarily utilized 16S rRNA sequencing. Therefore, future research should aim to validate the microbial functions and metabolic pathways through comprehensive metagenomic, transcriptomic, and metabolomic analyses, alongside enzyme activity assays. Additionally, larger-scale trials are necessary to substantiate the practical significance of these results in production settings.

## 5 Conclusion

The optimal supplementation levels of mannanase and Aspergillus co-fermented PKC were determined to be 0.5 and 0.1%, respectively. Under these specified conditions, the combined co-fermentation process significantly enhanced the nutritional profile of PKC while reducing the concentration of mannan. The incorporation of FPKC into the diet did not exert a significant effect on the production performance of beef cattle. During prolonged feeding, the diet containing FPKC improved the cattle’s ability to degrade protein, fat, and fiber in the feed, thereby enhancing nutrient digestibility. Concurrently, due to the adaptation of rumen microorganisms to the diet, there was a modification in the microbial community of beef cattle fed the FPKC diet, characterized by decreased levels of butyric acid and NH_3_-N, although there was no impact on the production of MCP and TVFA. Overall, the enzyme-bacteria co-fermentation of PKC did not adversely affect the growth performance of beef cattle and positively contributed to the improvement of nutrient digestibility. These findings provide a theoretical foundation and practical support for the application of FPKC in beef cattle diets.

## Data Availability

The data presented in this study are publicly available. The data can be found here: https://www.ncbi.nlm.nih.gov/sra, accession PRJNA1294514.

## Footnotes

1
https://qiime2.org
