# Supplementary material for: Fermented palm kernel cake improves the nutrient degradation of beef cattle by modulating the rumen microbiota
Source: Front Microbiol. 2025 Dec 11;16:1712275. doi: 10.3389/fmicb.2025.1712275 (PMC12740237; doi:10.3389/fmicb.2025.1712275)
Supplement: Supplementary file 1 [file Table_1.docx]

**Table S1.** Ingredient and chemical composition of diet fed during the experiment (DM basis)

| Items | CON^1^ | FP^2^ |
| --- | --- | --- |
| Ingredients % |  |  |
| Rapeseed meal | 6.82 | 6.82 |
| Corn | 66.95 | 51.33 |
| Fermented Palm kernel cake | - | 16.83 |
| Soybean mea | 9.56 | 8.35 |
| Wheat bran | 10.00 | 10.00 |
| ^3^Premix | 6.67 | 6.67 |
| ^4^Chemical compositions, % |  |  |
| Dry matter | 95.73 | 95.66 |
| Crude protein | 21.41 | 21.28 |
| Ether Extract | 2.94 | 2.14 |
| Ash | 7.93 | 7.73 |
| Gross energy (MJ/kg) | 16.35 | 16.02 |
| Neutral detergent fiber | 35.79 | 45.67 |
| Acid detergent fiber | 13.25 | 33.15 |

^1^ CON = The control group fed the basal diet.

^2^ FP = The FPKC diet.

^3^ One kilogram of premix contained the following ：Cu 5m g，Fe 30mg，Mn 9 mg，Zn 20 mg，I 5 mg，Co 2 mg ,Se 2.5 mg.

^4^ Chemical compositions were all measured values

**Table S2.** Nutrient composition and mannan concentration of solid fermented PKC of mannanase

| Item^1^ | Mannanase proportion | | | | | | SEM | *P*-Value | | | |
| --- | --- | --- | --- | --- | --- | --- | --- | --- | --- | --- | --- |
|  | 0% | 0.1% | 0.2% | 0.3% | 0.4% | 0.5% |  | ANOVA | Linear | Quadratic |  |
| Water (%) | 30.20 | 33.10 | 30.83 | 32.01 | 31.43 | 31.22 | 1.36 | 0.304 | 0.495- | 0.765 |  |
| DM (%) | 67.60 | 64.57 | 66.81 | 65.40 | 65.70 | 65.93 | 1.41 | 0.326 | 0.785 | 0.858 |  |
| CP (%) | 15.95 | 17.72 | 18.34 | 17.10 | 19.23 | 19.69 | 1.73 | 0.774 | 0.145 | 0.091 |  |
| GE (MJ/kg) | 19.61 | 19.87 | 19.39 | 19.70 | 19.33 | 19.57 | 1.30 | 0.504 | 0.407 | 0.707 |  |
| NDF (%) | 78.71^a^ | 75.61^b^ | 74.10^b^ | 72.66^c^ | 78.21^a^ | 75.67^b^ | 1.64 | 0.022 | 0.526 | 0.076 |  |
| ADF (%) | 52.15 | 49.99 | 48.87 | 50.59 | 52.72 | 47.41 | 2.63 | 0.347 | 0.390 | 0.666 |  |
| mannan (μg/g) | 181.52^a^ | 125.27^b^ | 111.17^c^ | 102.05^bc^ | 89.20^d^ | 82.42^d^ | 4.80 | <0.001 | <0.001 | <0.001 |  |

^1^ DM: Dry matter; CP: Crude protein; GE: Gross energy; NDF: Neutral detergent fiber; ADF: Acid detergent fiber.

^a, b, c, d^ Mean values within a row with unlike superscript letters were significantly different (*P* < 0.05)

**Table S3.** Nutrient composition and mannan concentration of solid fermented PKC of *Aspergillus niger*

| Item^1^ | *Aspergillus niger* proportion | | | | | | SEM | *P*-Value | | |
| --- | --- | --- | --- | --- | --- | --- | --- | --- | --- | --- |
|  | 0% | 0.1% | 0.2% | 0.3% | 0.4% | 0.5% |  | ANOVA | Linear | Quadratic |
| Water (%) | 30.20 | 32.59 | 33.65 | 32.87 | 31.95 | 32.07 | 1.29 | 0.191 | 0.968 | 0.241 |
| DM (%) | 67.60 | 64.58 | 63.09 | 63.80 | 64.31 | 64.38 | 1.19 | 0.067 | 0.152 | 0.014 |
| CP (%) | 15.95^b^ | 19.71^a^ | 19.11^a^ | 18.89^a^ | 20.95^a^ | 20.25^a^ | 1.63 | 0.024 | 0.027 | 0.094 |
| GE (MJ/kg) | 19.61 | 19.71 | 19.96 | 19.25 | 19.46 | 19.49 | 0.28 | 0.288 | 0.273 | 0.539 |
| NDF (%) | 78.71 | 76.99 | 77.13 | 77.18 | 77.41 | 77.67 | 0.88 | 0.381 | 0.482 | 0.155 |
| ADF (%) | 52.15^a^ | 50.31^a^ | 46.52^b^ | 44.08^b^ | 50.49^a^ | 51.71^a^ | 2.03 | 0.06 | 0.824 | 0.006 |
| mannan (μg/g) | 181.52^a^ | 51.60^b^ | 52.97b | 58.58b | 57.82b | 55.02b | 5.62 | <0.001 | 0.006 | <0.001 |

^1^ DM: Dry matter; CP: Crude protein; GE: Gross energy; NDF: Neutral detergent fiber; ADF: Acid detergent fiber.

^a, b^ Mean values within a row with unlike superscript letters were significantly different (P < 0.05)
